# Five-Year Follow-Up of POLARIS-01 Phase II Trial: Toripalimab as Salvage Monotherapy in Chinese Patients With Advanced Melanoma

**DOI:** 10.1093/oncolo/oyae045

**Published:** 2024-03-28

**Authors:** Bixia Tang, Rong Duan, Xiaoshi Zhang, Shuikui Qin, Di Wu, Jing Chen, Hong Yao, Zhihong Chi, Jun Guo, Xieqiao Yan

**Affiliations:** Key Laboratory of Carcinogenesis and Translational Research (Ministry of Education/Beijing), Department of Melanoma and Sarcoma Oncology, Peking University Cancer Hospital and Institute, Beijing, People’s Republic of China; Key Laboratory of Carcinogenesis and Translational Research (Ministry of Education/Beijing), Department of Genitourinary Oncology, Peking University Cancer Hospital and Institute, Beijing, People’s Republic of China; Biotherapy Center, State Key Laboratory of Oncology in South China, Collaborative Innovation Center for Cancer Medicine, Sun Yat-sen University Cancer Center, Guangzhou, People’s Republic of China; Cancer Centre of Jinling Hospital, Nanjing University of Chinese Medicine and Nanjing Medical University, Nanjing, People’s Republic of China; Department of Tumor Center, The First Hospital of Jilin University, Changchun, People’s Republic of China; Institute of Radiation Oncology, Cancer Center, Union Hospital, Tongji Medical College, Huazhong University of Science and Technology, Wuhan, People’s Republic of China; Department of Cancer Biotherapy Center, Tumor Hospital of Yunnan Province & The Third Affiliated Hospital of Kunming Medical University and Yunnan Cancer Center, Kunming, People’s Republic of China; Key Laboratory of Carcinogenesis and Translational Research (Ministry of Education/Beijing), Department of Melanoma and Sarcoma Oncology, Peking University Cancer Hospital and Institute, Beijing, People’s Republic of China; Key Laboratory of Carcinogenesis and Translational Research (Ministry of Education/Beijing), Department of Genitourinary Oncology, Peking University Cancer Hospital and Institute, Beijing, People’s Republic of China; Key Laboratory of Carcinogenesis and Translational Research (Ministry of Education/Beijing), Department of Genitourinary Oncology, Peking University Cancer Hospital and Institute, Beijing, People’s Republic of China

**Keywords:** long-term, follow-up, toripalimab, Chinese, melanoma, PD-1

## Abstract

**Background:**

To investigate the efficacy and toxicity after long-term follow-up of anti-PD-1 antibody in advanced melanoma with predominantly acral and mucosal subtypes.

**Methods and Patients:**

In the POLARIS-01 phase II trial, 128 Chinese patients with advanced melanoma refractory to standard therapy received toripalimab until disease progression or unacceptable toxicity for ≤2 years. For those who progressed after discontinuation due to 2-year treatment completion, rechallenge was allowed. The primary objectives were safety and overall response rate (ORR).

**Results:**

As of February 8, 2021, ORR was 17.3% (95% CI: 11.2-25.0) evaluated by the independent radiologic review committee. The median overall survival (OS) for patients with known melanoma subtypes was 16.3 m for acral, 41.5 m for nonacral cutaneous, and 10.3 m for mucosal melanoma. Thereafter, the evaluation was continued by investigators. As of November 4, 2022, 5 years after the last enrollment, median duration of response was 15.6 months (range, 3.7-64.5+), median progression-free survival (PFS) was 3.5 months (95% CI, 2.2-5.3), and 60-month OS rate was 28.5% (95% CI: 20.2-37.2). Thirteen patients completed a 2-year treatment of toripalimab, with the subtypes of acral (2/13), non-acral cutaneous (4/13), mucosal (3/13) and unknown primary (4/13). Five patients were rechallenged. Four of them, all of whom were non-mucosal, completed the rechallenge course of 2 years with PFS ≥ 24 months.

**Conclusions:**

This is the largest prospective anti-PD-1 trial with mature data in advanced melanoma in China. Toripalimab demonstrated a manageable safety profile and durable clinical response in Chinese patients with metastatic melanoma who had failed in standard therapy. Immunotherapy seems less efficacious for long-term responders with mucosal primaries as rechallenge therapy.

## Teaser

This study investigated the efficacy and toxicity after long-term follow-up of anti-PD-1 antibodies in advanced melanoma with predominantly acral and mucosal subtypes.

**Implications for Practice**

This is the largest prospective anti-PD-1 trial with mature data on advanced melanoma in China. ORR, PFS, and OS of melanoma with various subtypes (acral, nonacral cutaneous, and mucosal), and the correlation with PD-L1 expression, TMB, and cancer driver gene mutations (*BRAF*, *KIT*, *NRAS*, and *NF1*) were investigated. It was also found that immunotherapy seems less efficacious for long-term responders with mucosal primaries as rechallenge therapy.

## Introduction

Anti-PD-1 immunotherapy, pembrolizumab, and toripalimab have been sequentially approved for patients with advanced melanoma refractory to standard therapy in China in 2018. Although anti-PD-1 antibody has proved efficacy and safety in Caucasians in long-term follow-up,^1,2^ there is little data cumulating for anti-PD-1 monotherapy for Asians except Keynote-151 with 3-year follow-up.^3^ For Asians, acral and mucosal subtypes account for 70% of melanoma.^4^ As these 2 subtypes own distinct biological behavior compared with non-acral cutaneous melanoma,^5,6^ toripalimab, and pembrolizumab achieved only overall response rate (ORR) of 16.7-17.3% and progression-free survival (PFS) of 2.8-3.4 months in POLARIS-01 Phase II Trial^7^ and phase1b Keynote-151 study^3,8^ in Chinese patients with melanoma, which is much worse than the ORR of 28%-40% and PFS of 8.4 months in similar trials containing non-acral cutaneous melanoma mainly.^1,2^

Here, we presented the post hoc 5-year follow-up results of toripalimab in the POLARIS-01 phase II trial, including outcomes by melanoma subtypes, BRAFV600 status, PD-L1 expression, and TMB. Outcomes are also presented for patients who completed 2 years of toripalimab treatment and rechallenged with toripalimab monotherapy.

## Patients and Methods

### Study Design and Participants

POLARIS-01 was a multicenter, open-label, phase II, single-arm trial (ClinicalTrial.gov Identifier: NCT03013101) that evaluated the safety and efficacy of toripalimab in previously treated Chinese patients with advanced melanoma. Eligible patients were ≥18 years of age with pathologically confirmed local advanced or metastatic melanoma and were previously treated with systemic therapy; had ≥1 measurable lesion per Response Evaluation Criteria in Solid Tumors, version 1.1 (RECIST v1.1); had an Eastern Cooperative Oncology Group (ECOG) performance status of 0 or 1, adequate organ and bone marrow function, and willing to provide a tumor biopsy sample for PD-L1 analysis. The key exclusion criteria included prior anti-PD-1, anti-PD-L1, or anti-PD-L2 therapy; active central nervous system metastases; other serious, uncontrollable concomitant diseases that may affect protocol compliance or interfere with the interpretation of results. Detailed methods have been described previously.^7^

The study protocol and all amendments were approved by the institutional ethics committee at each site. The trial was conducted in accordance with Good Clinical Practice guidelines and Declaration of Helsinki. All patients provided written informed consent.

### Treatment

Patients received intravenous toripalimab 3 mg/kg once every 2 weeks until disease progression, unacceptable toxicity, or voluntary withdrawal. The patients who had no disease progression could be treated with toripalimab for up to 24 months, and then discontinuation of study therapy might be considered. The patients with complete response (CR) (as determined by investigators) might be considered to discontinue toripalimab therapy after at least 24 weeks of treatment. For those patients who progressed after discontinuation due to 2-year treatment completion, the rechallenge of toripalimab was also allowed. These patients might receive toripalimab again when radiographically confirmed disease progression if they met the following conditions after discontinuation: no other antitumor therapy was given after the last dose of toripalimab; met all the safety indicators listed in the study’s inclusion criteria and did not meet all the safety indicators listed in the exclusion criteria. After 2 years of rechallenge, the charity drug supply was stopped, and the treatment was discontinued.

### Assessment and Endpoints

Imaging by computed tomography or magnetic resonance imaging was performed before treatment, then once every 8 weeks in the first year and once every 12 weeks in the second year, once every 16 weeks in the third year until disease progression. The response was assessed per both RECIST v1.1 and Immune-related Response Evaluation Criteria in Solid Tumors (irRECIST) by investigators. Adverse events (AEs) were monitored throughout the study and for at least 30 days after toripalimab discontinuation (90 days for serious AEs probably related to toripalimab). AEs were graded according to the National Cancer Institute Common Terminology Criteria for Adverse Events (CTCAE), version 4.0. All patients were followed for survival (once every 3 months in the first year after withdrawing from the study, then once every 6 months) until the patients died or withdrew informed consent, or the study completed, whichever occurs first.

PD-L1 expression was assessed centrally in archival or fresh tumor biopsy samples obtained from patients prior to treatment using PD-L1 immunohistochemistry with SP142 antibody.^9^ PD-L1 positivity was defined as the presence of membrane staining of any intensity in ≥1% of tumor cells.

The primary endpoints were safety and clinical efficacy by objective response rate (ORR) per RECIST v1.1 by the independent radiologic review committee (IRC). Secondary endpoints included disease control rate (DCR), duration of response (DOR), progression-free survival (PFS), and overall survival (OS). As of February 8, 2021, the IRC evaluation ended, and the evaluation was continued by investigators.

### Statistical Analysis

Post hoc analysis was applied to examine the efficacy of toripalimab with 5 years of follow-up. All patients accepted at least 1 dose of study treatment was included. The main analysis of OS was assessed by subtypes: acral, non-acral cutaneous, mucosal melanoma, and melanoma with unknown primary lesion and the stratified log-rank test was applied accordingly to estimate the KM curve and median survival time for each study group. The 95% confidence interval (CI) was estimated by the Brookmeyer–Crowley method using a log–log function transformation. Subgroup analyses by molecular subtypes were also obtained. The 95% CI of ORR and DCR were determined by Clopper and Pearson method. All analyses were performed with SAS version 9.4.

## Results

### Patient Baseline Characteristics

A total of 128 patients with melanoma were enrolled in the POLARIS-01 trial. All received ≥1 dose of study treatment. Detail baseline characteristics have been reported previously^7^ and shown in Supplementary Table S1. The average age was 52.5 years (range, 21-76), 55.5% (*n* = 71) of patients were female, 42.5% (*n* = 54) had an ECOG performance status of 1, 28.3% (*n* = 36) had stage M1c disease, 19.7% had liver metastases (30.8% PD-L1 positive patients and 17.9% PD-L1 negative patients with liver metastases, respectively), 20.5% (*n* = 26) were identified as PD-L1 positive, and 26.8% (*n* = 34) had BRAF mutant. Most patients (39.4%; *n* = 50) had acral melanoma, 22.8% (*n* = 29) had non-acral cutaneous melanoma, 17.3% (*n* = 22) had mucosal melanoma, and melanoma subtype was unknown for 20.5% (*n* = 26). In addition, previous studies indicated the mutually exclusive mutations among BRAF, NRAS, and NF1.^10-12^ In terms of 10 patients with KIT mutation in the present study, 2 had NF1 mutations, while neither of them had BRAF and NRAS mutations. Median follow-up duration was 16.9 months (range, 0.9-70.1). Of 128 patients treated, 13 (10.2%) completed 2-year treatment, with the subtypes of acral (2/13), non-acral cutaneous (4/13), mucosal (3/13), and unknown primary (4/13).

### Safety

**Figure 1.**
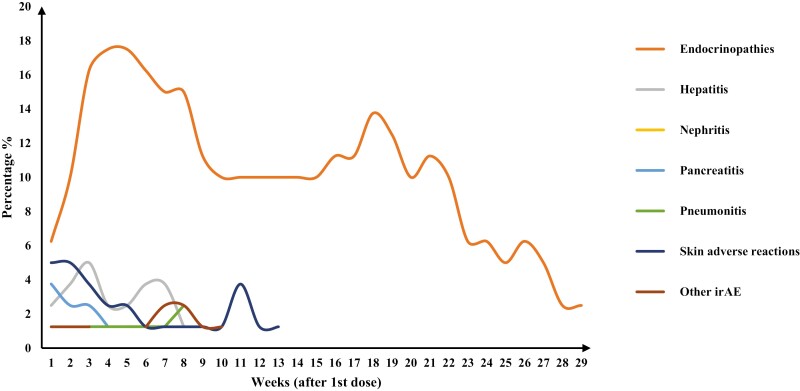
The approximate proportion of patients who developed immune-related AE since the first dose, defined as all records of each type of immune-related AE (the same patient may have multiple AE records).

The safety profile remained similar to that described previously.^7^ Immune-mediated endocrinopathy is the most common and long-lasting AE, which peaked in the fifth week after treatment initiation. No new safety signals were detected (Fig. 1) with longer follow-up.

### Efficacy

As of February 8, 2021, median PFS was 3.6 months (95% CI, 2.7-5.3), ORR was 17% (95% CI, 11.2-25.0; 0 CR/22 PR), and DCR was 57.5% (95% CI, 48.4-66.2; 51 SD) evaluated by IRC (Table 1). As mentioned above, the IRC evaluation ended on February 8, 2021, and the evaluation was continued by investigators. As of November 4, 2022, among the intention-to-treat (ITT) population (*n* = 127), the median DOR was 15.6 months (range, 3.7-64.5+) and median PFS was 3.5 months (95% CI, 2.2-5.3) evaluated by the investigator. As of the data cutoff, 85 (66.9%) patients had died, and the median OS was 20.0 months (95% CI, 14.8-29.3) (Table 1) with a 60-month OS rate of 28.5%.

**Table 1.** Clinical efficacy evaluated per RECIST v1.1 criteria.

	No.	ORR (%)^a^ (95% CI)	PFS (m)^a^ (95% CI)	OS (m) (95% CI)
Total	127	17.3 (11.2, 25.0)	3.6 (2.7, 5.3)	20.0 (14.8, 29.3)
Melanoma subtypes				
Acral	50	14.0 (5.8, 26.7)	3.2 (1.8, 3.7)	16.3 (10.9, 27.4)
Mucosal	22	0	2.1 (1.8, 5.3)	10.3 (6.6, 16.5)
Nonacral	29	31.0 (15.3, 50.8)	5.5 (1.9, 19.2)	41.5 (15.3, NE)
Unknown	26	23.1 (9.0, 43.6)	7.3 (3.5, 15.2)	33.9 (19.5, NE)
Molecular subtypes				
PD-L1 (–)	84	11.9 (5.9, 20.8)	2.9 (1.8, 3.6)	14.4 (10.8, 17.8)
PD-L1 (+)	26	38.5 (20.2, 59.4)	15.2 (3.6, NE)	63.3 (29.3, NE)
** **TMB_**low**_**(TMB < 3.6)**	**80**	**13.8 (7.1, 23.3)**	**3.6 (2.1, 5.3)**	**22.0 (15.3, 32.5)**
** **TMB_**high**_**(TMB ≥ 3.6)**	**31**	**22.6 (9.6, 41.1)**	**3.5 (1.8, 9.3)**	**16.0 (10.8, 33.9)**
BRAF (+)	34	32.4 (17.4, 50.5)	5.3 (3.5, 16.4)	63.3 (22.2, NE)
BRAF (–)	86	9.3 (4.1, 17.5)	3.3 (1.8, 3.6)	15.7 (11.7, 23.2)
NRAS (+)	16	6.2 (0.2, 30.2)	1.8 (1.7, 3.5)	16.2 (5.0, 19.5)
NRAS (–)	82	18.3 (10.6, 28.4)	3.7 (3.5, 5.5)	27.4 (16.3, 42.5)
NF1 (+)	10	20.0 (2.5, 55.6)	2.7 (1.7, 25.5)	12.6 (4.3, NE)
NF1 (–)	88	15.9 (9.0, 25.2)	3.6 (3.3, 5.5)	27.1 (16.9, 37.9)
KIT (+)	10	20.0 (2.5, 55.6)	5.3 (1.6, NE)	20.9 (7.6, 48.2)
KIT (–)	88	15.9 (9.0, 25.2)	3.5 (2.9, 5.5)	22.2 (16.0, 37.2)

^a^The ORR and PFS were evaluated by IRC.

Abbreviations: DCR, disease control rate; NE, not evaluable; ORR, objective response rate; PD-L1, programmed death ligand 1; RECIST v1.1, response evaluation criteria in solid tumors, version 1.1.

ORR by melanoma subtype was 14% for patients with acral melanoma, 31% for patients with non-acral cutaneous melanoma, 0% for patients with mucosal melanoma, and 23% for patients with unknown melanoma subtype. Median PFS was 3.2 months, 5.5 months, and 2.1 months in patients with acral melanoma, non-acral cutaneous melanoma, and mucosal melanoma, respectively. Median OS was 16.3 months in patients with acral melanoma, 41.5 months in patients with nonacral cutaneous melanoma, 10.3 months in patients with mucosal melanoma, and 33.9 months in patients with unknown melanoma subtypes (Table 1).

ORR was 39% (95% CI, 20.2-59.4) in PD-L1–positive patients, and 12% (95% CI, 5.9-20.8) in patients with PD-L1-negative disease. Median PFS and OS were 15.2 months (95% CI, 3.6-NE) vs 2.9 months (95% CI, 1.8-3.6) and 63.3 months (95% CI, 29.3-NE) vs 14.4 months (95% CI, 10.8-17.8) in patients with PD-L1-positive and PD-L1-negative disease, respectively (Table 1).

ORR was 14% (95% CI, 7.1-23.3) in TMB_low_ (TMB < 3.6) patients, and 23% (95% CI, 9.6-41.1) in TMB_high_ (TMB ≥ 3.6) patients. Median PFS and OS were 3.6 months (95% CI, 2.1-5.3) vs 3.5 months (95% CI, 1.8-9.3) and 22.0 months (95% CI, 15.3-32.5) vs 16.0 months (95% CI, 10.8-33.9) in patients with TMB_low_ and TMB_high_ disease, respectively (Table 1 and Supplementary Table S1). The top 20% TMB value in each subtype as cutoff for efficacy analysis has been reported in detail in the Supplementary Material to the previously published article.^7^

ORR was 9% (95% CI, 4.1-17.5) in patients with BRAF wild-type disease (BRAF−) and 32% (95% CI, 17.4-50.5) in patients with BRAF-mutant disease (BRAF+). Median PFS and OS were 3.3 months (95% CI, 1.8-3.6) vs. 5.3 months (95% CI, 3.5-16.4) and 15.7 months (95% CI, 11.7-23.2) vs 63.3 months (95% CI, 22.2-NE) in patients with BRAF-wild and BRAF-mutant disease, respectively (Table 1). ORR, median PFS, and OS by KIT, NRAS, and NF1 mutation status are listed in Table 1.

### Discontinuation of Treatment and Rechallenge

A total of 13 patients stopped receiving medication after 2 years of treatment. Five patients received a rechallenge of toripalimab at progression after initial SD/PR/CR. Of them, 2 were non-acral cutaneous, 1 each for unknown primary, mucosal, and acral, respectively. Four of the patients achieved PR and completed the rechallenge course (Table 2; Supplementary Fig. S1). For the 3 patients with mucosal melanoma, their PFS of the initial toripalimab therapy was 29, 35, and 39 months, respectively. Only one of them was rechallenged with toripalimab monotherapy and achieved SD for 11 months. For the 4 patients with non-acral cutaneous melanoma, 2 was rechallenged and achieved PR and the other 2 patients were still free of any anti-tumor therapy.

**Table 2.** Follow-up treatment and survival of patients who discontinued the investigational drug after 2 years of treatment.

ID	Melanoma subtype	Progression	Time to progression (m)	Following therapy	Rechallenge PFS (m)	OS ^a^(m)
S01036	Unknown	Y	40	Rechallenge	26+	69+
S01044	Non-acral cutaneous	Y	40	Rechallenge	26+	69+
S01109	Non-acral cutaneous	Y	34	Rechallenge	26+	63+
S01059	Acral	Y	37	Rechallenge	25	67+
S01049	Mucosal	Y	39	Rechallenge	11	46
S01075	Unknown	Y	25	Chemotherapy	NA	64
S01047	Mucosal	Y	29	NA	NA	44
S01100	Acral	Y	36	PD-1 comb. therapy	NA	63+
S02015	Unknown	Y	62	NA	NA	63+
S04001	Mucosal	Y	35	PD-1 comb. therapy	NA	59
S01039	Non-acral cutaneous	N	68+	No	–	69+
S01104	Non-acral cutaneous	N	62+	No	–	63+
S01066	Unknown	N	65+	No	–	66+

^a^The cutoff date was November 4, 2022.

Abbreviations: comb., combinational therapy; NA, not available.

## Discussion

Herein we presented the most mature data on the safety and efficacy of anti-PD-1 monotherapy as salvage therapy (≥second line) for Chinese patients with advanced melanoma.

The safety profile of toripalimab in the current study was similar to that observed in the earlier analysis of POLARIS-1^7^ and was consistent with the known safety profile of pembrolizumab in KEYNOTE-151.^3^ In this study, we further reported the phase of occurrence of different irAEs of toripalimab monotherapy. Endocrine-related (36.0%) and skin-related (32.4%) AEs were the majority of long-term irAEs involving immunotherapy.^13^ As shown in Fig. 1, immune-mediated endocrinopathy is the most common and long-lasting AE, which peaked at the fifth week. The most common endocrinopathy AE was hypothyroidism, hyperthyroidism, and thyroiditis.^7^ At the 18th week, new immune-related endocrinopathy occurs, including hypophysitis and diabetes mellitus.^7^ The patterns of endocrinopathy AEs involving the distinct organs were in line with the other studies.^14^ Cutaneous irAEs have a variety of clinical presentations, including eczematous, morbilliform, and lichenoid dermatoses, as well as vitiligo and pruritus.^15^ In our study, immune-related cutaneous AEs at early weeks and second peak are rash and pruritus, respectively. The occurrence of immune-mediated hepatitis was up to 5.0% at third week. And the second peak of 3.75% appeared at 6.5 weeks. The incidence of immune-mediated hepatitis was higher in this study than ever reported pembrolizumab or nivolumab in global trials.^2,16^ While the immune-mediated alanine aminotransferase increases and bilirubin increase were comparable with that in KEYNOTE-151 (23.3%).^3^

Previously, we have reported that the ORR was 17% and the DCR was 57.5% as of August 15, 2019, with a median follow-up of 16.4 months.^7^ As of February 8, 2021, the ORR remained at 17% and the DCR remained at 57.5%. After 5 years since the last enrollment, the median OS was 20.0 months and the 60-month OS rate was 28.5%, suggesting the durable efficacy of toripalimab in some patients. KEYNOTE-151 study, in which pembrolizumab acted as second-line therapy for Chinese melanoma patients, achieved a median OS of 13.2 months and a 36-month OS rate of 22.3%.^3^ Melanoma subtypes might not explain the disparity of survival benefits of the 2 trials well, as the proportion of the acral and mucosal subtypes were similar in POLARIS-1 and KEYNOTE-151 (39.4% vs 37.9% for acral, 17.3% vs 14.6% for mucosal). It might be attributed to other baseline characteristics, such as fewer patients with M1c stage (28.3% vs 53.4%) and liver metastasis (19.7% vs 25.2%) in POLARIS-1 than in KEYNOTE-151. Metastasis involving lymph nodes and/or lungs has a better response to anti-PD-1 monotherapy, while the liver is considered an immune evasion organ and responds poorly to immunotherapy alone.^17,18^

We further investigate the activity of toripalimab in subgroups of patients with tumors which have wild-type BRAF kinase vs patients with tumors having mutant BRAF. In the current study, patients with BRAFmut (26.8%) had an ORR of 32%, PFS of 5.3 months and OS of 63.3 months, while patients with BRAFwt (73.2%) had an ORR of 9%, PFS of 3.3 months and OS of 15.7 months. Larkin et al conducted a pooled analysis of 440 patients with unresectable stage III or stage IV melanoma treated with nivolumab. In patients evaluable for response, the ORR was 35% vs 30% in 217 BRAF^wt^ and 74 BRAF^mut^ patients. The ORR was 33% vs 25% in BRAFmut patients with no prior BRAF inhibitor therapy and with a prior BRAF inhibitor.^19^ Puzanov et al pooled analysis of clinical trials KEYNOTE-001, 002, and 006, in which pembrolizumab served as first-line or salvage therapy for unresectable melanoma. The ORR was 40% (447/1124) vs 34% (149/434), the 4-year PFS rate was 22.9% vs 19.8%, and the 4-year OS rate was 37.5% vs 35.1%, respectively for patients with BRAF WT and BRAF V600E/K-mutant melanoma.^20^ For 434 patients with BRAFmut, the ORR was 28% (77/271) vs 44% (72/163), the 4-year PFS rate was 15.2% vs 27.8%, and the 4-year OS rate was 26.9% vs 49.3%, respectively in BRAFmut patients with prior BRAF inhibitor therapy and without prior BRAF inhibitor.^20^ BRAF mutations occurred at significantly lower frequencies in acral (7/35, 20%) and mucosal (1/13, 7.7%) melanomas than non-acral cutaneous melanomas (10/17, 58.8%) in POLARIS-1, which were in line with the previous reports in Asians.^4^ We thought the lower efficacy of toripalimab in BRAFwt was related to the melanoma subtypes but not the drug itself. As BRAF and MEK inhibitors were not commercially available in China during the enrollment of POLARIS-1, only 1 of 34 patients with documented BRAF mutations was treated with vemurafenib in a previous clinical trial. For patients with BRAF mutant while without prior BRAF inhibitor, ORRs were similar among toripalimab, nivolumab, and pembrolizumab (32% vs 33% vs 44%).^19,20^

Although the optimal treatment duration remains to be defined, it is now universally accepted that for long-term responders, anti-PD-1-containing immunotherapy could be administered for 2 years and then withheld for active surveillance.^21,22^ For these patients with disease progression later, rechallenge is suggested to be considered because it might provide additional disease control*.*^23^ In accordance with prior studies,^24,25^ our study showed that for non-acral cutaneous melanoma (4 patients), immunotherapy monotherapy provides long-term efficacy (2 patients were at stable), and durable response at rechallenge course. However, immunotherapy seems less efficacious for long-term responders with mucosal primaries as rechallenge therapy. All 3 patients with mucosal melanoma relapsed and had with shorter OS than those patients with the other subtypes. Allison Betof Warner et al retrospectively revealed that patients with mucosal or acral primaries were less likely to have a CR compared to cutaneous primaries.^24^ Although the patient number was limited, our data prospectively provided some clue that for long-term responders other than non-acral cutaneous melanoma or unknown primary melanoma, discontinuation after 2-year administration should be cautious. For these subtypes, escalation therapy (PD-1 inhibitors combined with additional agents) rather than PD-1 inhibitor monotherapy rechallenge should be preferred.

This post hoc exploratory analysis of the long follow-up of the POLARIS-01 phase II trial suggested the safety and efficacy of toripalimab for Chinese patients with advanced melanoma. A phase III randomized trial comparing toripalimab monotherapy versus dacarbazine as first-line therapy for advanced melanoma in China has just been finished enrollment, which would further provide solid data on the safety and clinical efficacy of toripalimab (NCT03430297).

## Supplementary Material

oyae045_suppl_Supplementary_Tables_1

oyae045_suppl_Supplementary_Figures_1

## Data Availability

The data underlying this article will be shared on reasonable request to the corresponding author.
