# Supplementary material for: Five-Year Follow-Up of POLARIS-01 Phase II Trial: Toripalimab as Salvage Monotherapy in Chinese Patients With Advanced Melanoma
Source: Oncologist. 2024 Mar 28;29(6):e822–7. doi: 10.1093/oncolo/oyae045 (PMC11144968; doi:10.1093/oncolo/oyae045)
Supplement: oyae045_suppl_Supplementary_Tables_1 [file oyae045_suppl_supplementary_tables_1.docx]

Table S1. Patient characteristics.

| **Melanoma sub-category** | **Non-acral cutaneous (N=29)** | **Acral (N=50)** | **Mucosal (N=22)** | **Unknown primary (N=26)** | **Total (N=127)** | |
| --- | --- | --- | --- | --- | --- | --- |
| **Prior lines of treatment, N (%)** | | | | | |  |
| **1** | 8 (27.59) | 13 (26.00) | 9 (40.91) | 10 (38.46) | 40 (31.50) | |
| **2** | 10 (34.48) | 14 (28.00) | 3 (13.64) | 4 (15.38) | 31 (24.41) | |
| **3+** | 11 (37.93) | 23 (46) | 10 (45.45) | 12 (46.15) | 56 (44.09) | |
| **ORR, %, (95% CI) *** | | | | | |  |
| **PD-L1 (+)** | 66.67 (29.93,92.51) | 0 | 0 | 33.33 (9.92,65.11) | 38.5 (20.2, 59.4) | |
| **PD-L1 (-)** | 13.33 (1.66,40.46) | 14.63 (5.57,29.17) | 0 | 18.18 (2.28,51.78) | 11.9 (5.9, 20.8) | |
| **TMB_high_ (≥3.6)** | 40 (5.27,85.34) | 22.22 (2.81,60.01) | 0 | 40 (5.27,85.34) | 22.6 (9.6, 41.1) | |
| **TMB_low_ (＜3.6)** | 27.78 (9.69,53.48) | 6.67 (0.82,22.07) | 0 | 18.75 (4.05,45.65) | 13.8 (7.1, 23.3) | |
| **BRAF (+)** | 62.5 (24.49,91.48) | 22.22 (2.81,60.01) | 0 | 26.67 (7.79,55.1) | 32.4 (17.4, 50.5) | |
| **BRAF (-)** | 15 (3.21,37.89) | 10.53 (2.94,24.8) | 0 | 10 (0.25,44.5) | 9.3 (4.1, 17.5) | |
| **NRAS (+)** | 33.33 (0.84,90.57) | 0 | 0 | 0 | 6.2 (0.2, 30.2) | |
| **NRAS (-)** | 30 (11.89,54.28) | 12.5 (3.51,28.99) | 0 | 27.78 (9.69,53.48) | 18.3 (10.6, 28.4) | |
| **NF1 (+)** | 0 | 0 | 0 | 100 (15.81,100) | 20.0 (2.5, 55.6) | |
| **NF1 (-)** | 31.82 (13.86,54.87) | 11.11 (3.11,26.06) | 0 | 15.79 (3.38,39.58) | 15.9 (9.0, 25.2) | |
| **KIT (+)** | 0 | 16.67 (0.42,64.12) | 0 | 50 (1.26,98.74) | 20.0 (2.5, 55.6) | |
| **KIT (-)** | 30.43 (13.21,52.92) | 9.09 (1.92,24.33) | 0 | 21.05 (6.05,45.57) | 15.9 (9.0, 25.2) | |
| **PFS, m, (95% CI) *** | | | | | |  |
| **PD-L1 (+)** | 40.0 (1.7, NE) | NE (5.3, NE) | 3.6 (NE, NE) | 5.5 (1.4, 62.0) | 15.2 (3.6, NE) | |
| **PD-L1 (-)** | 2.4 (1.8, 6.3) | 1.9 (1.8, 3.7) | 1.8 (1.7, 5.3) | 7.3 (1.6,10.9) | 2.9 (1.8, 3.6) | |
| **TMB_high_ (≥3.6)** | 6.3 (1.9, NE) | 3.5 (1.6, NE) | 1.8 (NE, NE) | NE (1.4, NE) | 3.5 (1.8, 9.3) | |
| **TMB_low_ (＜3.6)** | 5.5 (1.8, NE) | 2.1 (1.8, 3.7) | 2.7 (1.7, NE) | 5.4 (1.8, 10.9) | 3.6 (2.1, 5.3) | |
| **BRAF (+)** | 16.4 (1.8, NE) | 3.5 (1.6, 5.4) | NE (5.3, NE) | 7.3 (1.6, 25.5) | 5.3 (3.5, 16.4) | |
| **BRAF (-)** | 3.5 (1.8, 19.2) | 2.1 (1.8, 5.3) | 1.8 (1.7, 35.0) | 5.5 (1.4, NE) | 3.3 (1.8, 3.6) | |
| **NRAS (+)** | 6.3 (1.9, NE) | 1.7 (1.7, 3.5) | 1.8 (1.7, NE) | 3.5 (1.4, NE) | 1.8 (1.7, 3.5) | |
| **NRAS (-)** | 5.5 (1.8, 40.0) | 3.5 (1.8, 5.3) | 3.6 (1.7, NE) | 7.3 (3.5, 15.2) | 3.7 (3.5, 5.5) | |
| **NF1 (+)** | 3.5 (NE, NE) | 1.8 (1.8, NE) | 1.8 (1.7, NE) | 18.2 (10.9, NE) | 2.7 (1.7, 25.5) | |
| **NF1 (-)** | 5.9 (1.9, 40.0) | 3.3 (1.8, 5.3) | 3.6 (0.9, NE) | 5.3 (1.8, 7.8) | 3.6 (3.3, 5.5) | |
| **KIT (+)** | 3.6 (1.6, NE) | 10.9 (NE, NE) | NA | NE (1.8, NE) | 5.3 (1.6, NE) | |
| **KIT (-)** | 5.5 (2.9, 40.0) | 2.1 (1.8, 3.7) | 1.8 (1.7, NE) | 5.3 (1.8, 7.8) | 3.5 (2.9, 5.5) | |
| **OS, m, (95% CI) *** | | | | | |  |
| **PD-L1 (+)** | NE (5.8, NE) | NE (NE, NE) | NE (1.7, NE) | 64.0 (5.1, NE) | 63.3 (29.3, NE) | |
| **PD-L1 (-)** | 18.8 (6.9, NE) | 14.0 (10.8, NE) | 9.7 (4.9, 16.2) | NE (8.2, NE) | 14.4 (10.8, 17.8) | |
| **TMB_high_ (≥3.6)** | 18.8 (6.9, NE) | NE (1.4, NE) | 14.8 (NE, NE) | NE (13.5, NE) | 16.0 (10.8, 33.9) | |
| **TMB_low_ (＜3.6)** | NE (15.7, NE) | 13.7 (10.1, NE) | 11.7 (4.3, 44.0) | 64.0 (13.5, NE) | 22.0 (15.3, 32.5) | |
| **BRAF (+)** | NE (22.2, NE) | NE (8.9, NE) | NE (16.5, NE) | 64.0 (9.5, NE) | 63.3 (22.2, NE) | |
| **BRAF (-)** | 18.8 (8.8, NE) | 16.0 (10.8, NE) | 10.3 (6.6, 16.2) | 23.2 (8.2, NE) | 15.7 (11.7, 23.2) | |
| **NRAS (+)** | 18.8 (6.9, NE) | 10.9 (3.9, NE) | 6.6 (1.7, NE) | NE (19.5, NE) | 16.2 (5.0, 19.5) | |
| **NRAS (-)** | NE (15.7, NE) | NE (10.8, NE) | 13.7 (4.3, 44.0) | 64.0 (13.5, NE) | 27.4 (16.3, 42.5) | |
| **NF1 (+)** | 11.7 (NE, NE) | 14.9 (13.7, NE) | 7.1 (4.3, NE) | 64.0 (NE, NE) | 12.6 (4.3, NE) | |
| **NF1 (-)** | NE (18.8, NE) | 17.8 (10.7, NE) | 16.2 (1.8, NE) | NE (13.5, NE) | 27.1 (16.9, 37.9) | |
| **KIT (+)** | NE (7.6, NE) | NE (NE, NE) | NA | 11.2 (7.6, NE) | 20.9 (7.6, 48.2) | |
| **KIT (-)** | NE (15.7, NE) | 16.9 (10.7, NE) | 12.6 (4.3, 44.0) | 64.0 (13.5, NE) | 22.2 (16.0, 37.2) | |

*Calculated based on evaluable patients. NA indicated no PFS/OS event occurred in the corresponding subgroups. NE, not evaluable.

Table S2. Distribution of irAE within each melanoma subgroup.

| **irAE** | **Non-acral cutaneous (N=29)** | **Acral**  **(N=50)** | **Mucosal**  **(N=22)** | **Unknown primary**  **(N=26)** |
| --- | --- | --- | --- | --- |
| **Endocrine disorders** | 11 (37.93%) | 9 (18%) | 4 (18.18%) | 6 (23.08%) |
| **Eye disorders** | 2 (6.90%) | 0 | 0 | 0 |
| **Gastrointestinal disorders** | 0 | 2 (4%) | 0 | 1 (3.85%) |
| **Hepatobiliary disorders** | 2 (6.90%) | 3 (6%) | 0 | 1 (3.85%) |
| **Investigations** | 0 | 0 | 0 | 3 (11.54%) |
| **Metabolism and nutrition disorders** | 0 | 1 (2%) | 1 (4.55%) | 0 |
| **Respiratory, thoracic and mediastinal disorders** | 1 (3.45%) | 1 (2%) | 0 | 0 |
| **Skin and subcutaneous tissue disorders** | 1 (3.45%) | 2 (4%) | 0 | 5 (19.23%) |
