# Supplementary material for: Five-Year Follow-Up of POLARIS-01 Phase II Trial: Toripalimab as Salvage Monotherapy in Chinese Patients With Advanced Melanoma
Source: Oncologist. 2024 Mar 28;29(6):e822–7. doi: 10.1093/oncolo/oyae045 (PMC11144968; doi:10.1093/oncolo/oyae045)
Supplement: oyae045_suppl_Supplementary_Figures_1 [file oyae045_suppl_supplementary_figures_1.pptx]

## Slide 1
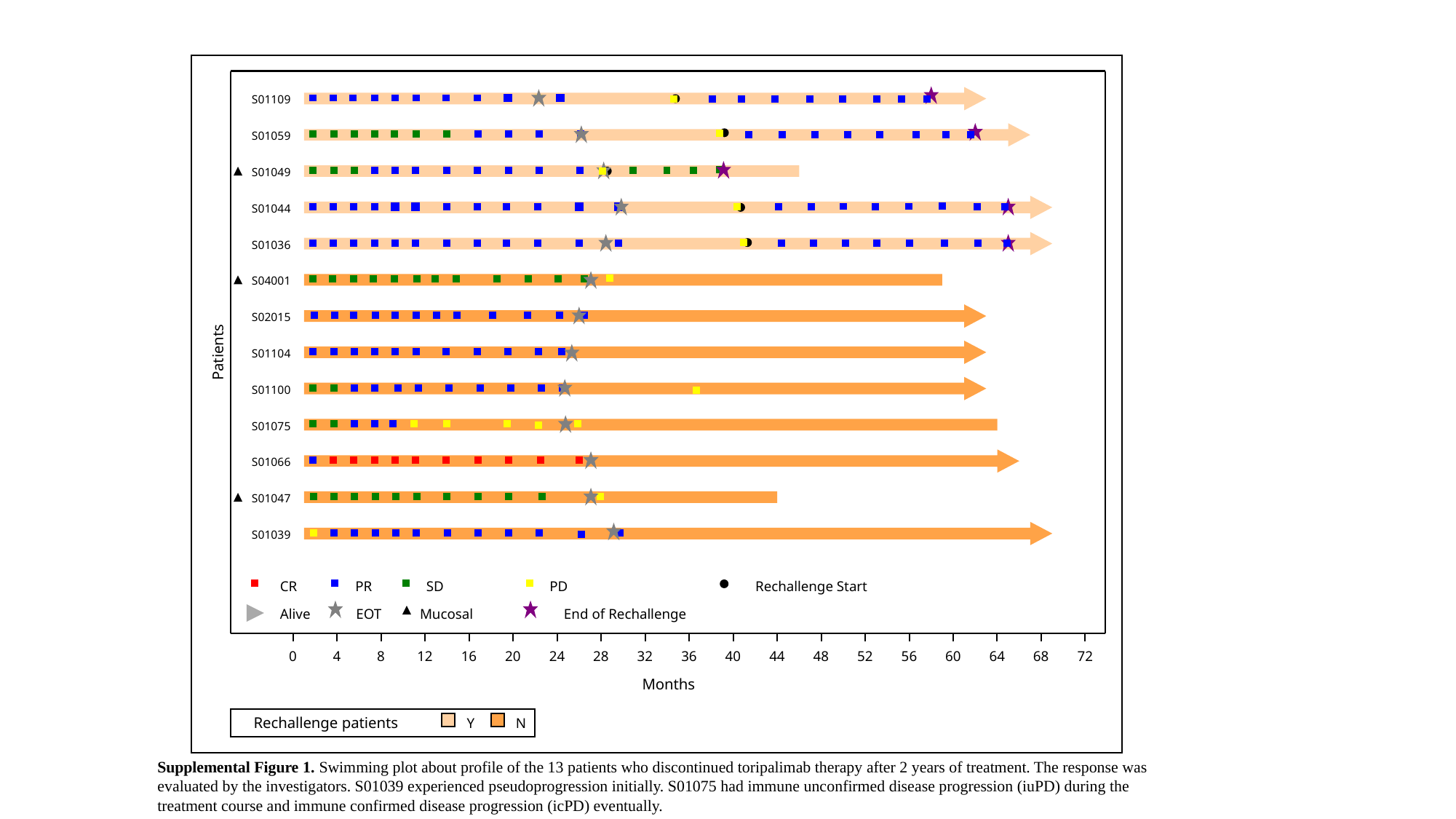

Patients
0
4
8
12
16
20
24
28
32
36
40
44
48
52
56
60
64
68
72
Months
S01109
S01059
S01049
S01044
S01036
S04001
S02015
S01104
S01100
S01075
S01066
S01047
S01039
CR
PR
SD
PD
Rechallenge Start
Alive
EOT
Mucosal
End of Rechallenge
Rechallenge patients
Y
N
Supplemental Figure 1. Swimming plot about profile of the 13 patients who discontinued toripalimab therapy after 2 years of treatment. The response was evaluated by the investigators. S01039 experienced pseudoprogression initially. S01075 had immune unconfirmed disease progression (iuPD) during the treatment course and immune confirmed disease progression (icPD) eventually.
